# Thirty-five years of evolving inflammatory paradigms in sepsis (1991–2025): a comprehensive mapping of immune dysregulation research, anti-inflammatory targets, and translational barriers

**DOI:** 10.3389/fimmu.2026.1892665

**Published:** 2026-09-21

**Authors:** Hao-Han Rao, Feng Guo, Jie Tian, Chun-Jian Zuo, Huan-Wen Chen

**Affiliations:** Department of Thoracic Surgery, The First Affiliated Hospital of Chongqing Medical University, Chongqing, China

**Keywords:** anti-inflammatory therapeutics, global mapping, immune dysregulation, NLRP3 inflammasome, regulated cell death, Sepsis

## Abstract

**Background:**

Sepsis represents a life-threatening dysregulation of the host inflammatory response, progressing from innate immune hyperactivation and pro-inflammatory cytokine release to a secondary immunosuppressive phase. Although the diagnostic framework has evolved from systemic inflammatory response syndrome (SIRS)-based criteria to the organ dysfunction-centric Sepsis-3 definition, the longitudinal evolution of inflammatory research paradigms, emerging anti-inflammatory targets, and barriers limiting clinical translation has not been systematically characterized.

**Methods:**

Peer-reviewed publications (n=106,884; 1991-2025) were retrieved from PubMed and the Web of Science Core Collection. A temporally stratified computational framework analyzed publication dynamics, keyword co-occurrence, citation bursts, and thematic evolution across sequential diagnostic epochs. Co-cited references were graded by Oxford Centre for Evidence-Based Medicine (OCEBM) criteria to evaluate evidence maturation and translational readiness.

**Results:**

Annual publications grew from 974 (1991) to 8,492 (2025; *R*^2^ = 0.9841). The United States led in publication volume (32,406), while the Netherlands (77.58 citations/publication) and France (76.99) recorded the highest citation impact. The co-citation evidence base transitioned from expert consensus (Level 5) toward randomized controlled trials (RCTs) (Level 1) and prospective cohorts (Level 2). Keyword burst and thematic evolution analyses identified three sequential inflammatory research phases: early dominance of pro-inflammatory mediators (lipopolysaccharide, TNF-α), an intermediate shift toward immunosuppression and organ dysfunction, and a contemporary frontier centered on NLRP3 inflammasome signaling, regulated cell death (RCD) pathways, and gut-lung axis modulation. Emerging computational approaches, including AI-driven predictive models, demonstrated increasing research momentum; however, limited external validation, infrastructure requirements, and interpretability challenges remain barriers to clinical translation.

**Conclusions:**

This comprehensive mapping reveals a fundamental transition in global sepsis research, from broad cytokine suppression toward precision immunomodulation of specific immune dysregulation mechanisms, while highlighting persistent challenges in translating emerging discoveries into clinical practice. Future research should prioritize mechanistically targeted interventions and equitable implementation of anti-inflammatory strategies across diverse healthcare settings.

## 1 Introduction

Sepsis represents the most severe clinical manifestation of a dysregulated host inflammatory response to infection, culminating in life-threatening organ dysfunction (1). Data from the Global Burden of Diseases study indicate that sepsis accounts for approximately 20% of total global mortality annually, with the highest epidemiological burden concentrated in resource-limited regions (2). Despite iterative refinements of the Surviving Sepsis Campaign (SSC) guidelines, sepsis remains a leading cause of intensive care unit (ICU) mortality and long-term morbidity worldwide (3, 4), underscoring the persistent gap between advances in inflammatory biology and bedside clinical outcomes. Although substantial progress has been achieved in elucidating cytokine networks, immune dysregulation, and molecular mechanisms underlying sepsis progression, translating these biological discoveries into effective, targeted clinical interventions remains challenging.

The past 35 years have witnessed a fundamental reconceptualization of sepsis, driven by evolving insights into the immunopathological mechanisms underlying its progression. The inaugural 1991 consensus (Sepsis-1) characterized the syndrome through the lens of systemic inflammatory response syndrome (SIRS), emphasizing generalized pro-inflammatory activation as the primary pathophysiological driver (5, 6). The landmark Sepsis-3 definition (2016) challenged this framework by reframing sepsis as infection-induced organ dysfunction, operationalized by an increase of two or more points in the Sequential Organ Failure Assessment (SOFA) score (7). This conceptual shift acknowledged the inadequacy of SIRS criteria in capturing the heterogeneous, organ-specific nature of immune dysregulation. This transition drove a substantial expansion of the scientific literature, shifting investigative focus from macroscopic syndrome characterization and clinical management toward molecular dissection of immune heterogeneity and precision immunomodulation. Emerging research directions have increasingly incorporated multi-omics profiling, gut-organ inflammatory interactions, and regulated cell death (RCD) pathways, including pyroptosis, ferroptosis, and cuproptosis, reflecting a broader transition from generalized inflammatory suppression toward mechanism-guided therapeutic strategies (8–11).

At the molecular level, this paradigm shift reflects deepening insight into the cell signaling cascades governing the septic inflammatory response. The SIRS framework relied on macroscopic physiological markers without capturing the underlying molecular complexity. Subsequent research has elucidated the central role of innate immune pattern recognition—mediated by Toll-like receptors and the NLR family pyrin domain containing 3 (NLRP3) inflammasome—in amplifying the host inflammatory response through pathogen-associated molecular pattern (PAMP) and damage-associated molecular pattern (DAMP) signaling (12–14). Downstream caspase-1 activation drives the maturation and secretion of pro-inflammatory cytokines IL-1β and IL-18, while gasdermin D-mediated pyroptosis sustains the systemic inflammatory cascade through lytic cell death (13, 15). Equally important, the clinical course of sepsis is shaped not by hyperinflammation alone, but by a concurrent immunosuppressive phase characterized by lymphocyte apoptosis, monocyte deactivation, and impaired antigen presentation, which renders patients susceptible to secondary infections and perpetuates organ dysfunction (12, 16, 17). This dynamic balance between pro-inflammatory activation and immune paralysis has reframed sepsis as a disorder of dysregulated host immunity, redirecting therapeutic development toward precision immunomodulation at specific inflammatory checkpoints.

The rapid diversification of sepsis research, further accelerated by the COVID-19 pandemic—which established viral-induced cytokine storm and immune dysregulation as a parallel sepsis-like paradigm (18, 19) —has made it increasingly difficult for clinicians and researchers to identify actionable research priorities. Although prior quantitative evaluations have mapped inflammatory sub-domains such as nanomedicine-based anti-inflammatory strategies (20), sepsis-associated acute kidney injury (SA-AKI) (21), mitochondrial-driven immune dysfunction (22), and gut microbiota dysbiosis (23), these investigations predominantly rely on static, retrospective citation analyses. Recent mechanistic reviews have further emphasized the central role of cytokine networks in sepsis-associated systemic inflammation, immune dysregulation, and multiple organ dysfunction, highlighting the complexity of inflammatory responses beyond isolated cytokine pathways (24). These studies do not fully capture the dynamic thematic transitions—particularly the ongoing shift toward precision immunomodulation—that characterize the contemporary Sepsis-3 era (25). Furthermore, descriptive metadata mapping has not been applied to systematically trace the structural evolution of the inflammatory knowledge base across sequential diagnostic epochs, or to identify the translational barriers impeding the clinical deployment of emerging immunotherapeutic strategies.

To address these gaps, the present study applies a temporally stratified computational framework to a comprehensive dataset of 106,884 peer-reviewed publications spanning 35 years (1991–2025), with four primary objectives: (1) to quantify global publication outputs and international collaborative networks shaping the inflammatory sepsis research landscape; (2) to map the structural evolution of the empirical knowledge base across major inflammatory paradigm shifts, from SIRS-centric consensus to organ dysfunction-directed precision immunology; (3) to delineate emergent thematic trajectories and citation momentum driving precision diagnostics, targeted immunomodulation, and predictive analytics; and (4) to construct a clinical translational matrix evaluating core inflammatory research domains against evidence hierarchies and implementation barriers. This synthesis aims to translate macro-scale literature dynamics into strategic recommendations for inflammatory research investment, international collaboration, and equitable resource allocation (**Figure 1**).

**Figure 1:**
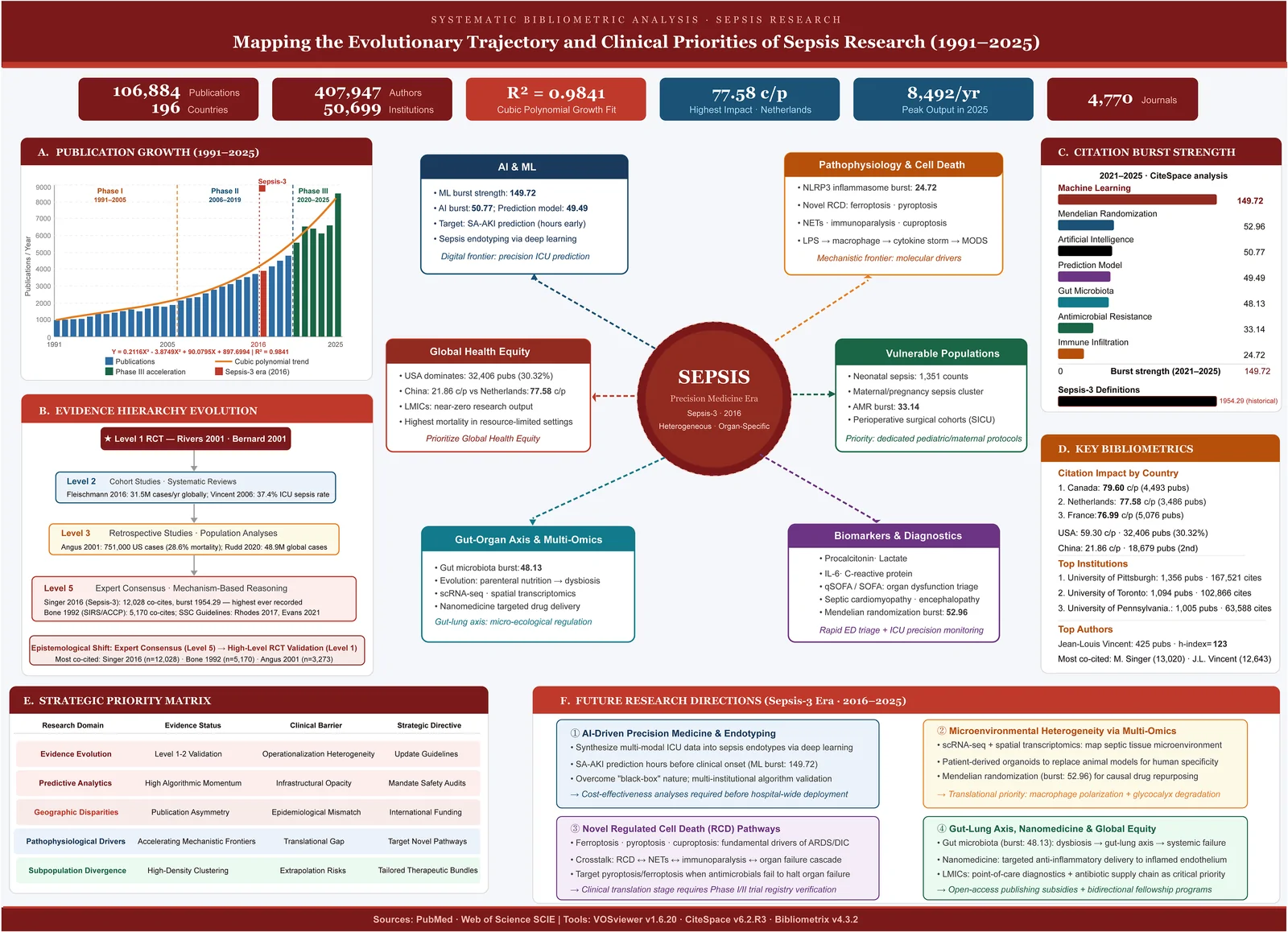
Mapping the evolutionary trajectory and clinical priorities of sepsis research (1991-2025). **(A)** Publication growth trajectory. Illustrates the three growth phases of annual publication output from 1991 to 2025. **(B)** Evidence hierarchy evolution. Illustrates the distribution and evolution of identified evidence levels, highlighting the transition from expert consensus toward higher-level empirical validation. (Central hub) The precision sepsis ecosystem. Depicts the transition from Sepsis-3–defined immune dysregulation toward six emerging research domains encompassing molecular mechanisms, predictive analytics, and translational strategies. **(C)** Citation burst strength (2021-2025). Highlights the top emerging research topics and keywords ranked by burst strength. **(D)** Key bibliometrics. Summarizes the leading countries, institutions, and authors by citation impact and productivity. **(E)** Strategic matrix. Maps global sepsis research domains across qualitative evidence status, clinical barriers, and strategic directives. **(F)** Future research directions. Outlines four primary focal points for upcoming sepsis research and global equity. AI, artificial intelligence; AMR, antimicrobial resistance; ARDS, acute respiratory distress syndrome; c/p, citations per publication; DIC, disseminated intravascular coagulation; EGDT, early goal-directed therapy; ICU, intensive care unit; LMIC, low- and middle-income country; ML, machine learning; MODS, multiple organ dysfunction syndrome; NETs, neutrophil extracellular traps; NLRP3, NLR family pyrin domain containing 3; qSOFA, quick sequential organ failure assessment; RCD, regulated cell death; RCT, randomized controlled trial; SA-AKI, sepsis-associated acute kidney injury; scRNA-seq, single-cell RNA sequencing; SICU, surgical intensive care unit; SIRS, systemic inflammatory response syndrome; SOFA, sequential organ failure assessment; SSC, surviving sepsis campaign.

## 2 Methods

### 2.1 Data source, information retrieval, and screening strategy

A systematic electronic search was conducted on April 20, 2026, across PubMed and the Web of Science Core Collection (Science Citation Index Expanded, SCIE) to retrieve peer-reviewed publications on sepsis-related inflammatory research published between January 1, 1991, and December 31, 2025. The 1991 chronological baseline was deliberately aligned with the inaugural Sepsis-1 consensus definition (5, 6), enabling comprehensive coverage of the field’s conceptual trajectory from SIRS-centric inflammatory criteria through to the organ dysfunction-directed Sepsis-3 framework. Publications indexed in 2026 were excluded to preserve full-year data integrity for longitudinal trend modeling. Searches were executed using Title/Abstract fields in PubMed and Title/Abstract/Author Keywords in SCIE, utilizing structured Boolean search strings detailed in **Supplementary Figure 1**. The initial combined retrieval yielded 264,134 records (PubMed: 128,002; SCIE: 136,132).

Search criteria were designed to construct a clinically relevant bibliometric dataset focused on human sepsis-related inflammatory research. Inclusion was restricted to original research articles and review articles published in English. Non-peer-reviewed content—including meeting abstracts, editorials, letters, and book chapters—was excluded. To focus the dataset on clinically translatable inflammatory pathways, NOT Boolean operators were applied to systematically exclude preclinical animal studies, isolated cell line experiments, and *in vitro*-only investigations. Publications originating from non-clinical disciplines, including veterinary medicine, zoological sciences, and plant biology, were additionally omitted.

Literature records were independently reviewed by two investigators to ensure dataset eligibility and methodological consistency. Classification discrepancies were resolved through consensus discussion, with unresolved disagreements adjudicated by the corresponding author. Following the removal of duplicate entries, non-conforming document types, and non-English publications, a highly curated dataset comprising 106,884 unique, peer-reviewed clinical publications (consisting of 93,054 original articles and 13,830 review articles as data sources) was finalized for subsequent structural analysis (**Supplementary Figure 1**).

### 2.2 Computational mapping and stratified analytical framework

To integrate longitudinal publication trends with granular contemporary insights, analyses were conducted within a temporally stratified, multi-dimensional framework using CiteSpace (v.6.2.R3), VOSviewer (v.1.6.20), and the Bibliometrix R-package (v.4.3.2). Global collaborative structures—encompassing countries, institutions, journals, and annual publication volumes—were mapped across the full 35-year spectrum (1991–2025) to characterize the foundational architecture of inflammatory sepsis scholarship. Thematic quadrant modeling and knowledge evolution pathway analyses were restricted to the post-2016 Sepsis-3 era to minimize semantic interference from obsolete SIRS-based terminology and ensure accurate characterization of contemporary inflammatory research priorities.

VOSviewer was applied to construct and visualize complex bibliometric networks (26), including: (i) collaborative mapping of countries, institutions, journals, and individual researchers, with Hirsch’s h-index employed as a standardized metric of investigator scientific impact (27); (ii) co-citation analysis of publications and references grounded in Small’s co-citation theory to delineate the intellectual architecture and foundational knowledge structures of inflammatory sepsis research (28); and (iii) keyword co-occurrence network construction, wherein node size reflects cumulative publication frequency and color-coding identifies thematic clusters or temporal evolutionary shifts in research priorities.

CiteSpace provided complementary insights into citation dynamics and interdisciplinary knowledge flows (29, 30): (i) dual-map overlay analysis was applied to visualize citation trajectories across academic disciplines, tracing translational pathways from molecular immunology toward clinical implementation; and (ii) citation burst detection, implemented via Kleinberg’s burst-detection algorithm (29, 31), identified keywords and references with statistically significant increases in scholarly attention, thereby identifying emerging inflammatory research frontiers and major shifts in investigative focus.

The Bibliometrix R-package, accessed via the Biblioshiny web interface (32, 33), was employed to: (i) map geographical distributions of publication outputs and characterize global research hotspots; and (ii) conduct longitudinal thematic map analysis and thematic evolution modeling to track the consolidation, divergence, and emergence of inflammatory research themes across the three sequential sepsis definition epochs.

### 2.3 Statistical modeling, cross-database validation, and evidence grading

#### 2.3.1 Statistical modeling

Annual publication growth was modeled using polynomial regression analysis in Microsoft Excel 2021, with goodness-of-fit assessed by the coefficient of determination (*R*^2^) to characterize the structural growth trajectory of inflammatory sepsis research over the 35-year study period.

#### 2.3.2 Cross-database sampling validation

In compliance with computational analyses of publicly available bibliographic data, a prospective cross-database validation study was conducted prior to final dataset consolidation. A stratified random sample of 500 records was systematically drawn from the pooled pre-deduplication retrieval of 264,134 records, proportionally stratified across three predefined temporal epochs corresponding to the major sepsis definition milestones: the Sepsis-1 era (1991–2001; n = 75), the Sepsis-2 era (2002–2015; n = 175), and the Sepsis-3 era (2016–2025; n = 250). This stratified design exceeds the statistically required minimum sample of 385 records (95% confidence interval; 5% margin of error; conservative p = 0.5 assumption) while ensuring proportional temporal representation across the full study period.

Each sampled record was independently verified by two investigators, who confirmed bibliographic accuracy and correct database attribution in both PubMed and SCIE. Discordant records were adjudicated by the corresponding author. An inter-database concordance rate of 98.4% (492/500 records confirmed) was observed, exceeding the pre-specified acceptability threshold of ≥ 95%, confirming the integrity of the consolidated dataset prior to bibliometric analysis (**Supplementary Figure 1**).

#### 2.3.3 Evidence-level grading

To objectively evaluate the translational maturation of the inflammatory sepsis knowledge base, foundational co-cited references driving major paradigm shifts were extracted from the co-citation network and independently graded by two authors according to the Oxford Centre for Evidence-Based Medicine (OCEBM) criteria (34), mapping evidence levels from expert consensus (Level 5) through systematic reviews and meta-analyses to large-scale randomized controlled trials (RCTs) (Level 1). Grading discrepancies were resolved by consensus with the corresponding author. This framework provides a reproducible, externally anchored benchmark for assessing the translational readiness of key inflammatory research domains identified through computational mapping.

All visualization parameters were standardized across analytical platforms to ensure graphical reproducibility and cross-tool comparability (**Supplementary Figure 1**).

## 3 Results

### 3.1 Quantitative analysis of publications

Global sepsis research output increased continuously from 1991 to 2025, with annual publication volumes fitting a cubic polynomial regression model (*R***^2^ = **0.9841) (**Figure 2A**). This trajectory comprises three distinct temporal phases. Phase I (1991-2005) represented initial growth, with annual publications rising from 974 to 1,875. Phase II (2006-2019) showed steady expansion, with annual outputs increasing from over 2,000 to 4,800. Phase III (2020-2025) exhibited accelerated growth, with annual publications reaching 8,492 by 2025, corresponding to a cumulative total of 380,201 citations.

**Figure 2:**
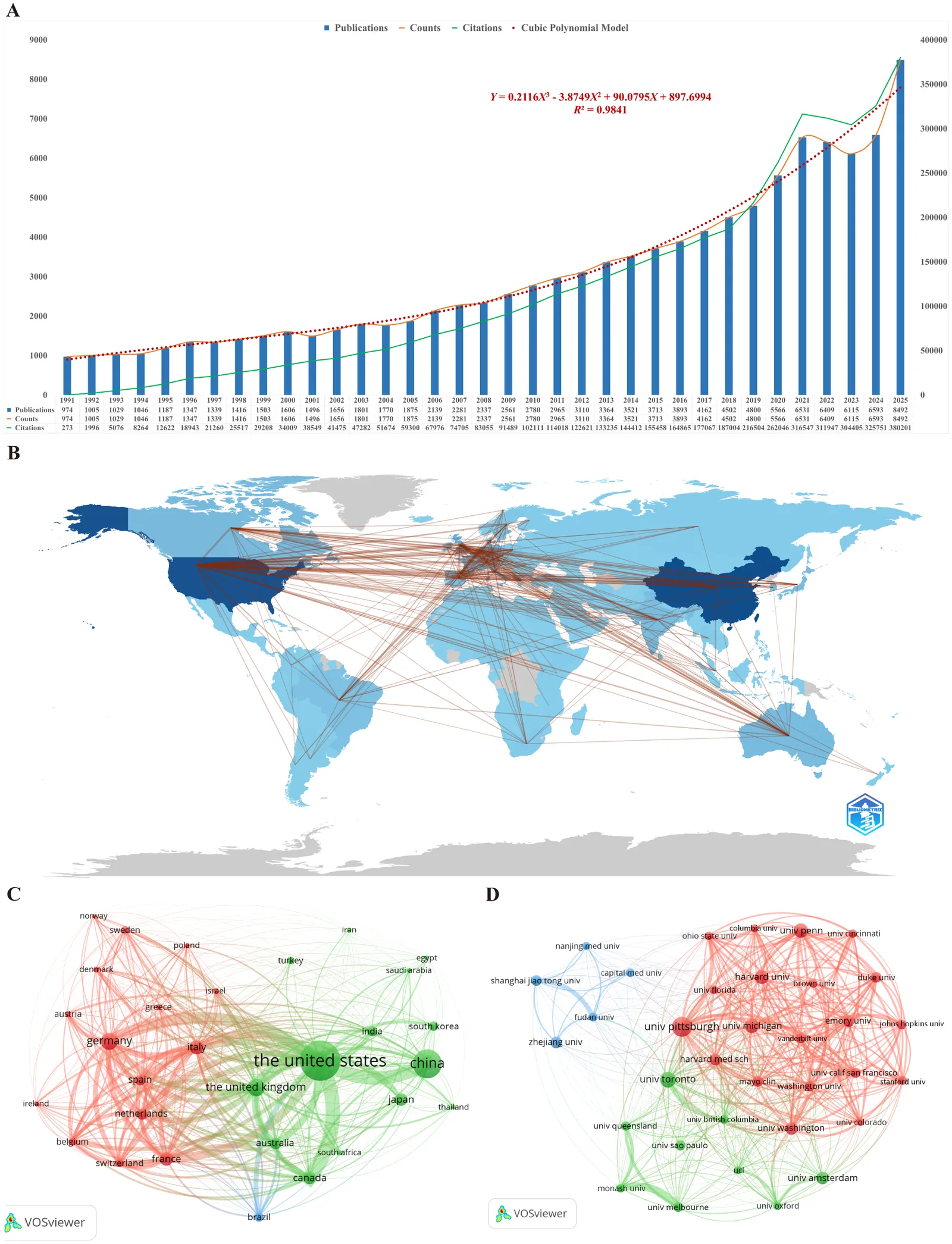
The visualization of sepsis research. **(A)** Annual research output on the research of sepsis. The orange curve indicates the annual publication trend. The green curve indicates the annual citation trend. The red dotted trend line is fitted to the number of publications using a cubic polynomial model. **(B)** The geographical spread of contributions, where darker colors indicate higher publication volumes. **(C)** Cooperation network of different countries. In this network, node size reflects the number of publications per country, different colors represent distinct collaboration clusters, and line thickness indicates the strength of international collaboration. This study enrolled 30 countries based on minimum number of documents of a country equal to 700 (attraction/repulsion values: 2/1). **(D)** The visualization of institutions on the sepsis fields. This study selected 34 institutions based on the minimum number of publications equal to 550 for visualization, and constructed a collaborative network based on the number and relationship of publications of each institution (attraction/repulsion values: 4/-1.

### 3.2 Geographical and institutional distribution

The United States recorded the highest contribution with 32,406 publications and 1,921,734 citations (**Figures 2B, C**; **Supplementary Table 1**). China ranked second in output with 18,679 publications. Canada (79.60 citations per article), the Netherlands (77.58), and France (76.99) recorded higher average citation impact than China (21.86). European nations, including the United Kingdom and Germany, maintained stable citation rates and consistent publication volumes throughout the study period.

Institutional output remains concentrated within the United States (**Figure 2D**; **Supplementary Table 1**). Leading domestic institutions by publication volume included the University of Pittsburgh with 1,356 publications and 167,521 citations, the University of Pennsylvania with 1,005 publications and 63,588 citations, and the University of Michigan with 984 publications and 83,358 citations, followed by Harvard University, the University of Washington, Harvard Medical School, and Emory University. Prominent non-US entities included the University of Toronto with 1,094 publications and the University of Amsterdam with 880 publications. Within China, Zhejiang University with 796 publications and 19,216 citations represented the highest institutional output.

### 3.3 Dissemination channels and disciplinary interactions

*Critical Care Medicine* had the highest publication volume with 2,391 publications and 247,955 citations, followed by *PLOS ONE* and *Shock* (**Figure 3A**; **Supplementary Table 2**). *Intensive Care Medicine* (IF: 22.1) and *Critical Care* (IF: 9.3) were additional primary dissemination channels. Co-citation network analysis identified *Critical Care Medicine* as the primary foundational source with 154,264 co-citations, followed by general medical journals including the *New England Journal of Medicine* with an IF of 78.5, *JAMA* with an IF of 55, and the *Lancet* with an IF of 88.5 (**Figure 3B**; **Supplementary Table 2**). Dual-map overlay analysis characterized the structural flow of clinical knowledge (**Figure 4**). Green paths represent citation flows from the *Medicine/Medical/Clinical* domain toward *Molecular/Biology/Genetics* and *Health/Nursing/Medicine*. Orange paths represent citation flows from *Molecular/Biology/Immunology* toward *Molecular/Biology/Genetics* and *Health/Nursing/Medicine*.

**Figure 3:**
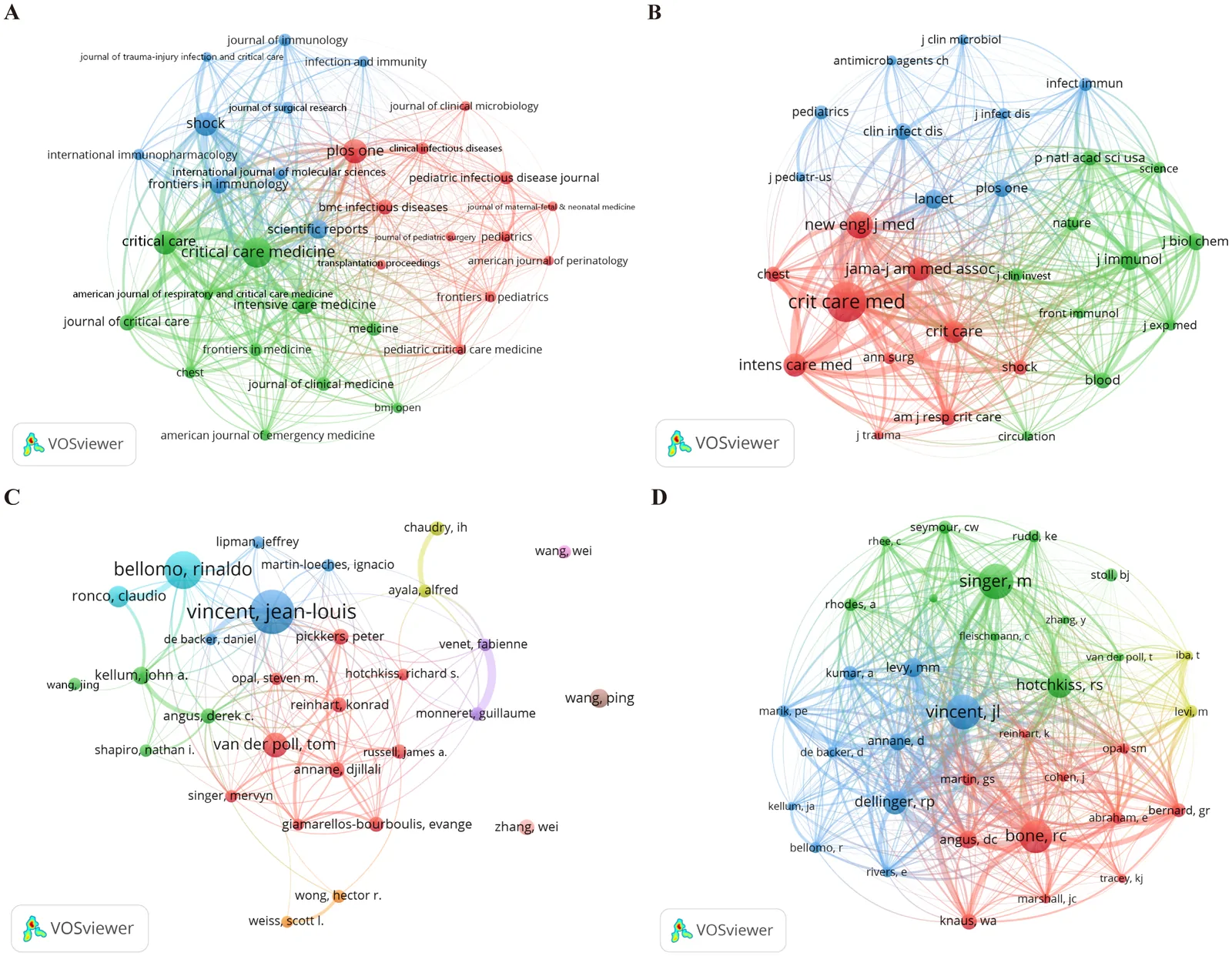
The visualization of journals, co-cited journals, authors and co-cited authors on research of sepsis. **(A)** This study enrolled 32 journals based on the minimum number of relevant publications equal to 350 and mapped the journal network (attraction/repulsion values: 2/-1). Node size represents publication volume, colors indicate journal clusters based on research focus similarity, and line thickness shows co-citation strength between journals. **(B)** More than 18,000 co-citation journals (29 journals) were filtered to map the co-citation network (attraction/repulsion values: 3/-1). Node size reflects total co-citations, different colors represent thematic clusters, and connecting lines indicate co-citation relationships with thickness proportional to co-citation frequency. **(C)** A collaborative network was constructed based on 29 researchers whose number of published documents is more than or equal to 100 (attraction/repulsion values: 1/-7). Node size corresponds to the number of publications per author, different colors represent collaborative clusters or institutional affiliations, and line thickness indicates the strength of co-authorship relationships. **(D)** This study selected 34 authors to map the co-citation network based on minimum co-citations equals to 2,000 (attraction/repulsion values: 1/-1). Node size reflects total co-citation frequency, colors distinguish different research communities or temporal periods, and connecting lines show co-citation relationships with thickness representing co-citation strength.

**Figure 4:**
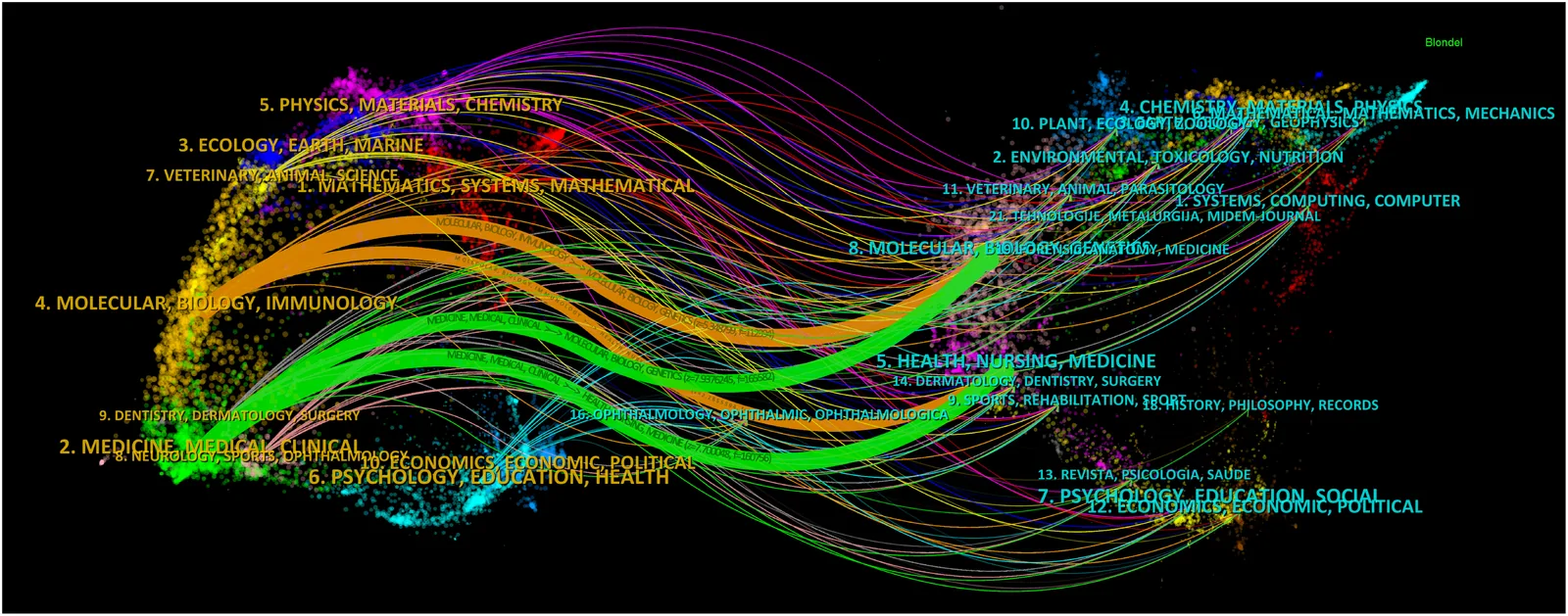
The dual-map overlay of journals on research of sepsis.

### 3.4 Author contribution and intellectual foundation

Jean-Louis Vincent had the highest publication output with 425 publications, 82,872 citations, and an h-index=123, followed by Rinaldo Bellomo with 353 publications and an h-index=87, and Tom Van Der Poll with 241 publications and an h-index=71 (**Figure 3C**; **Supplementary Table 3**). Co-citation analysis identified M. Singer with 13,020 co-citations, J. L. Vincent with 12,643 co-citations, and R. C. Bone with 11,635 co-citations as the most frequently co-cited authors in the field (**Figure 3D**; **Supplementary Table 3**), whose collective works span from the foundational SIRS consensus definitions to the contemporary Sepsis-3 framework.

### 3.5 Evidence maturation and structural knowledge shifts

Within the co-cited reference network, Singer et al. (2016, *JAMA*) recorded the highest citation count with 12,028 citations (7) and the strongest citation burst with a strength=1954.29. This was preceded by Bone et al. (1992, *Chest*) with 5,170 citations (6) and Angus et al. (2001, *Crit Care Med*) with 3,273 citations (35) and a burst strength = 314.41 (**Figures 5A, B**; **Table 1**). Chronologically, Bone et al. (1992) standardized the initial physiological parameters for SIRS (6); Angus et al. (2001) quantified the baseline public health burden in the United States with 751,000 annual cases and a 28.6% mortality rate (35); and Singer et al. (2016) defined sepsis as infection-induced organ dysfunction with a SOFA score increase of ≥ 2 points (7).

**Figure 5:**
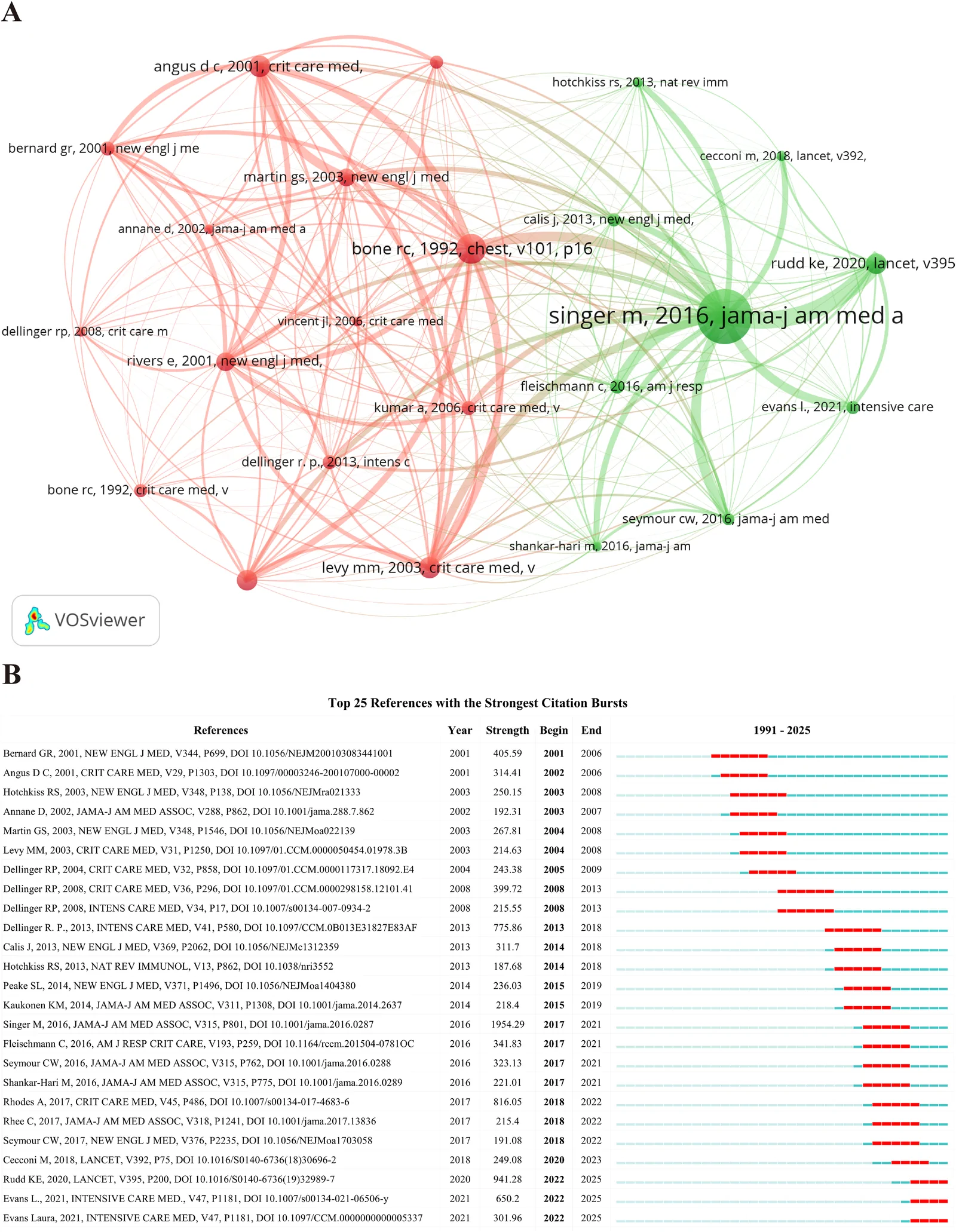
The visualization of co-cited references on research of sepsis. **(A)** A collaborative network was constructed based on 24 references whose number of co-citation is more than or equal to 1,000 (attraction/repulsion values: 1/-2). Node size represents total co-citation frequency, different colors indicate thematic clusters or research periods, and line thickness shows the strength of co-citation relationships between references. **(B)** Top 25 references with strong citation bursts. A red bar indicates high citations in that year, with bar width reflecting citation intensity and blue bars representing baseline citation periods.

**Table 1:** Characteristics and evidence classification of the top 20 co-cited references in sepsis research.

Rank	Primary reference	Total co-citations	Publication title	Bibliometric identifier	Study methodology	Evidence hierarchy (Level)^b^	Core clinical/technical impact	Reference
1	Singer et al. (2016)	12028	The Third International Consensus Definitions for Sepsis and Septic Shock (Sepsis-3)	singer m, 2016, jama-j am med assoc, v315, p801. doi:10.1001/jama.2016.0287	Expert opinion	Level 5	Redefined Sepsis as life-threatening organ dysfunction (SOFA score increase ≥ 2) and Septic Shock via vasopressor/lactate requirements; introduced qSOFA for bedside screening and retired the term “severe sepsis”.	(7)
2	Bone et al. (1992)**^a^**	5170	Definitions for sepsis and organ failure and guidelines for the use of innovative therapies in sepsis. The ACCP/SCCM Consensus Conference Committee. American College of Chest Physicians/Society of Critical Care Medicine	bone rc, 1992, chest, v101, p1644. doi:10.1378/chest.101.6.1644	Expert opinion	Level 5	Established the original standardized definitions for SIRS, Sepsis, Severe Sepsis, and Septic Shock; introduced the concept of MODS to unify clinical research and treatment.	(6)
3	Angus et al. (2001)	3273	Epidemiology of severe sepsis in the United States: analysis of incidence, outcome, and associated costs of care	angus dc, 2001, crit care med, v29, p1303. doi:10.1097/00003246-200107000-00002	Retrospective comparative studies	Level 3	Established that severe sepsis affects 751,000 people annually in the U.S. with a 28.6% mortality rate, identifying age as a primary driver of incidence and cost ($16.7 billion total).	(35)
4	Levy et al. (2003)	3194	2001 SCCM/ESICM/ACCP/ATS/SIS International Sepsis Definitions Conference	levy mm, 2003, crit care med, v31, p1250. doi:10.1097/01.ccm.0000050454.01978.3b	Expert opinion	Level 5	Expanded the clinical criteria for sepsis to reflect bedside experience while maintaining 1991 definitions; introduced the PIRO model as a framework for future research.	(36)
5	Rhodes et al. (2017)	3105	Surviving Sepsis Campaign: International Guidelines for Management of Sepsis and Septic Shock: 2016	rhodes a, 2017, crit care med, v45, p486. doi:10.1097/ccm.0000000000002255	Expert opinion	Level 5	Provided 93 clinical statements (32 strong) on early management and resuscitation of sepsis/septic shock using the GRADE system to assess evidence quality.	(37)
6	Rudd et al. (2020)	3043	Global, regional, and national sepsis incidence and mortality, 1990-2017: analysis for the Global Burden of Disease Study	rudd ke, 2020, lancet, v395, p200. doi:10.1016/s0140-6736(19)32989-7	Retrospective comparative studies	Level 3	Identified 48.9 million incident sepsis cases and 11.0 million deaths in 2017 (19.7% of all global deaths), revealing high regional burdens despite a global downward trend in age-standardized mortality since 1990.	(42)
7	Rivers et al. (2001)	2684	Early goal-directed therapy in the treatment of severe sepsis and septic shock	rivers e, 2001, new engl j med, v345, p1368. doi:10.1056/nejmoa010307	Randomized controlled trials (RCTs)	Level 1	Found that 6 hours of early goal-directed therapy prior to ICU admission significantly reduced in-hospital mortality (30.5% vs. 46.5%) and decreased the severity of organ dysfunction (APACHE II scores)	(49)
8	Martin et al. (2003)	2571	The epidemiology of sepsis in the United States from 1979 through 2000	martin gs, 2003, new engl j med, v348, p1546. doi:10.1056/nejmoa022139	Retrospective comparative studies	Level 3	Identified an 8.7% annualized increase in sepsis incidence (1979–2000) and a shift toward gram-positive and fungal pathogens; while the mortality rate fell from 27.8% to 17.9%, the total number of deaths continued to rise.	(43)
9	Kumar et al. (2006)	1895	Duration of hypotension before initiation of effective antimicrobial therapy is the critical determinant of survival in human septic shock	kumar a, 2006, crit care med, v34, p1589. doi:10.1097/01.ccm.0000217961.75225.e9	Retrospective comparative studies	Level 3	Established a linear relationship between antimicrobial delay and mortality, showing a 7.6% average decrease in survival for every hour of delay after the onset of septic shock-induced hypotension.	(44)
10	Bernard et al. (2001)	1864	Efficacy and safety of recombinant human activated protein C for severe sepsis	bernard gr, 2001, new engl j med, v344, p699. doi:10.1056/nejm200103083441001	Randomized controlled trials (RCTs)	Level 1	Found that treatment with drotrecogin alfa activated significantly reduced 28-day mortality (24.7% vs. 30.8%) in severe sepsis, though it was associated with an increased risk of serious bleeding (3.5% vs. 2.0%).	(50)
11	Fleischmann et al. (2016)	1851	Assessment of Global Incidence and Mortality of Hospital-treated Sepsis. Current Estimates and Limitations	fleischmann c, 2016, am j resp crit care, v193, p259. doi:10.1164/rccm.201504-0781oc	Systematic reviews of cohort studies	Level 2	Estimated an annual global burden of 31.5 million sepsis cases and 5.3 million deaths via meta-analysis, while identifying a critical lack of epidemiological data from low- and middle-income countries.	(47)
12	Dellinger et al. (2013)	1816	Surviving Sepsis Campaign: international guidelines for management of severe sepsis and septic shock, 2012	dellinger r. p., 2013, intens care med, v41, p580. doi:10.1007/s00134-012-2769-8	Expert opinion	Level 5	Standardized 93 recommendations including early quantitative resuscitation within 6h, antimicrobial administration within 1h, 30 mL/kg crystalloid bolus, norepinephrine as first-line vasopressor, and low tidal volume ventilation for ARDS.	(38)
13	Bone et al. (1992)**^a^**	1701	American-College Of Chest Physicians Society Of Critical Care Medicine Consensus Conference - Definitions For Sepsis And Organ Failure And Guidelines For The Use Of Innovative Therapies In Sepsis	bone rc, 1992, crit care med, v20, p864. doi:10.1097/00003246-199206000-00025	Expert opinion	Level 5	Established the original standardized definitions for SIRS, Sepsis, Severe Sepsis, and Septic Shock; introduced the concept of MODS and recommended severity scoring for mortality assessment.	(41)
14	Evans et al. (2021)	1642	Surviving sepsis campaign: international guidelines for management of sepsis and septic shock 2021	evans l., 2021, intensive care med., v47, p1181. doi:10.1007/s00134-021-06506-y	Expert opinion	Level 5	Updated 93 recommendations emphasizing capillary refill time-guided resuscitation, refined antimicrobial timing (within 1h for shock, 3h for possible sepsis), and a new focus on post-ICU care and long-term recovery.	(39)
15	Hotchkiss et al. (2003)	1631	The pathophysiology and treatment of sepsis	hotchkiss rs, 2003, new engl j med, v348, p138. doi:10.1056/nejmra021333	Mechanism-based reasoning	Level 5	Challenged the “uncontrolled inflammation” paradigm by highlighting the role of apoptosis and immune suppression in sepsis pathophysiology; advocated for therapies targeting both pro- and anti-inflammatory phases.	(12)
16	Seymour et al. (2016)	1554	Assessment of Clinical Criteria for Sepsis: For the Third International Consensus Definitions for Sepsis and Septic Shock (Sepsis-3)	seymour cw, 2016, jama-j am med assoc, v315, p762. doi:10.1001/jama.2016.0288	Retrospective comparative studies	Level 3	Validated SOFA as the best predictor of mortality in ICU settings and established qSOFA as a superior, simpler screening tool for non-ICU environments compared to SIRS or SOFA.	(45)
17	Hotchkiss et al. (2013)	1261	Sepsis-induced immunosuppression: from cellular dysfunctions to immunotherapy	hotchkiss rs, 2013, nat rev immunol, v13, p862. doi:10.1038/nri3552	Mechanism-based reasoning	Level 5	Defined sepsis as a dynamic balance between pro- and anti-inflammatory responses, proposing that future success lies in biomarker-guided immunotherapy tailored to the patient’s specific immune phase.	(16)
18	Cecconi et al. (2018)	1206	Sepsis and septic shock	cecconi m, 2018, lancet, v392, p75. doi:10.1016/s0140-6736(18)30696-2	Mechanism-based reasoning	Level 5	Summarized the Sepsis-3 transition to defining sepsis as infection with organ dysfunction (quantified by SOFA score), while highlighting the WHO’s global priority status and the push toward individualized, molecular-profile-based management.	(40)
19	Vincent et al. (2006)	1143	Sepsis in European intensive care units: results of the SOAP study	vincent jl, 2006, crit care med, v34, p344. doi:10.1097/01.ccm.0000194725.48928.3a	Prospective cohort studies	Level 2	Established a 37.4% sepsis incidence across European ICUs, identifying positive fluid balance and Pseudomonas infection as critical independent predictors of increased mortality.	(48)
20	Shankar-Hari et al. (2016)	1081	Developing a New Definition and Assessing New Clinical Criteria for Septic Shock: For the Third International Consensus Definitions for Sepsis and Septic Shock (Sepsis-3)	shankar-hari m, 2016, jama-j am med assoc, v315, p775. doi:10.1001/jama.2016.0289	Retrospective comparative studies	Level 3	Defined Septic Shock as a subset of sepsis with circulatory and metabolic abnormalities; established clinical criteria requiring both vasopressors (to maintain MAP ≥ 65 mmHg) and serum lactate > 2 mmol/L after fluid resuscitation, identifying a high-risk group with > 40% mortality.	(46)

ACCP, American College of Chest Physicians; SCCM, Society of Critical Care Medicine; ESICM, European Society of Intensive Care Medicine; ATS, American Thoracic Society; SIS, Surgical Infection Society; SIRS, Systemic Inflammatory Response Syndrome; MODS, Multiple Organ Dysfunction Syndrome; SOFA, Sequential [Sepsis-related] Organ Failure Assessment; qSOFA, quick Sequential Organ Failure Assessment; RCTs, Randomized controlled trials; ICU, Intensive Care Unit; ARDS, Acute Respiratory Distress Syndrome; WHO, World Health Organization; MAP, Mean Arterial Pressure; GRADE, Grading of Recommendations, Assessment, Development and Evaluations; APACHE II, Acute Physiology and Chronic Health Evaluation II; PIRO, Predisposition, Insult, Response, and Organ dysfunction; SOAP, Sepsis Occurrence in Acutely Ill Patients.

^a^Rank 2 and Rank 13 represent two simultaneously published versions of the same 1991 ACCP/SCCM Consensus Conference, appearing in Chest and Critical Care Medicine respectively.

^b^Evidence Hierarchy: The levels of evidence were classified according to the Levels of Evidence for Therapeutic Studies scale adapted from the Centre for Evidence-Based Medicine, as described by Burns et al. (2011). The hierarchy is defined as follows:

Level 1: Randomized controlled trials (RCTs) or systematic reviews of RCTs.

Level 2: Prospective cohort studies or systematic reviews of cohort studies.

Level 3: Case-control studies or retrospective comparative studies.

Level 4: Case series, or cohort/case-control studies of lower quality (e.g., no control group).

Level 5: Expert opinion or mechanism-based reasoning.

The evidence base was dominated by expert consensus and mechanism-based reasoning at Level 5 (n = 10) (6, 7, 12, 16, 36–41), primarily represented by the Sepsis-3 diagnostic definitions (7) and the international SSC guidelines (37, 39). Level 3 retrospective comparative studies (n = 6) (35, 42–46) followed, exemplified by the epidemiological work of Angus et al. (35) and Rudd et al. (42). Level 2 evidence (n = 2) was provided by Fleischmann et al. (47) and Vincent et al. (48). Level 1 evidence (n = 2) comprised two landmark RCTs: the early goal-directed therapy trial by Rivers et al. (49) and the efficacy assessment of recombinant human activated protein C by Bernard et al. (50). This distribution reflects a transition from consensus-derived frameworks toward empirical prospective validation.

### 3.6 Thematic hotspots and core research axes

The global sepsis research landscape, mapped across six thematic clusters, identified four primary research axes (**Figure 6A**; **Supplementary Table 4**). These include (1) pathophysiological mechanisms and systemic organ failure, which delineate the molecular cascades from localized cellular insult to SIRS and multiple organ dysfunction syndrome (MODS); (2) etiological landscapes and vulnerable cohorts, focusing on microbiological origins, neonatal or maternal sepsis, and antimicrobial resistance patterns; (3) clinical bedside management and biomarker-guided diagnostics, combining rapid emergency department triage with ICU-based organ support and outcome synthesis; and (4) digital frontiers and predictive analytics, utilizing machine learning (ML) algorithms for early complication detection and personalized treatment strategies.

**Figure 6:**
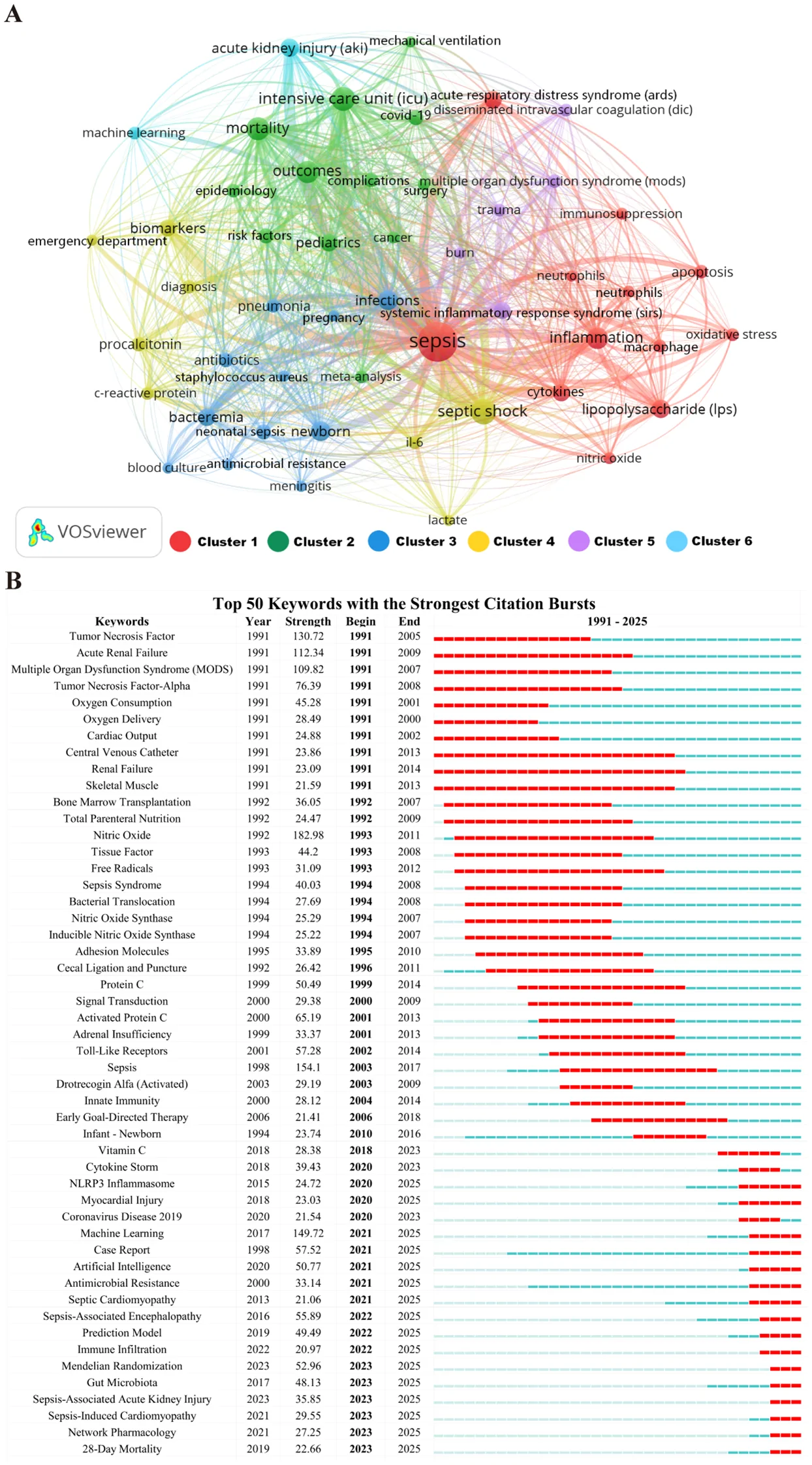
Keywords visualization analysis. **(A)** Keyword cluster analysis (network visualization). Node size reflects keyword frequency, different colors represent distinct research theme clusters, line thickness indicates co-occurrence strength between keywords, and spatial proximity shows thematic similarity. **(B)** Top 50 keywords with strongest citation bursts (100). Red bars indicate periods of increased citation activity (bursts), blue bars show baseline periods, and bar thickness represents burst strength intensity.

Between 2021 and 2025, research priorities shifted toward advanced predictive analytics and organ-specific pathology (**Figure 6B**). These hotspots focus on improving clinical outcomes, including 28-day mortality, using AI and ML models. Current research targets the pathophysiology of severe complications, including septic cardiomyopathy, sepsis-associated encephalopathy, and AKI. Concurrently, causal inference methodologies, including Mendelian randomization and network pharmacology, are increasingly applied to identify complex molecular drivers—notably the NLRP3 inflammasome, gut microbiota dysbiosis, and cell-specific immune infiltration—as emerging anti-inflammatory research targets (13, 51).

### 3.7 Temporal evolution and thematic development trajectory

Longitudinal thematic evolution analysis across three temporal intervals (**Figure 7A**) tracked the progression of research themes across the Sepsis-3 era. Overall, the analysis revealed a transition from macroscopic clinical management toward precision medicine and microenvironmental research between 2016 and 2025.

**Figure 7:**
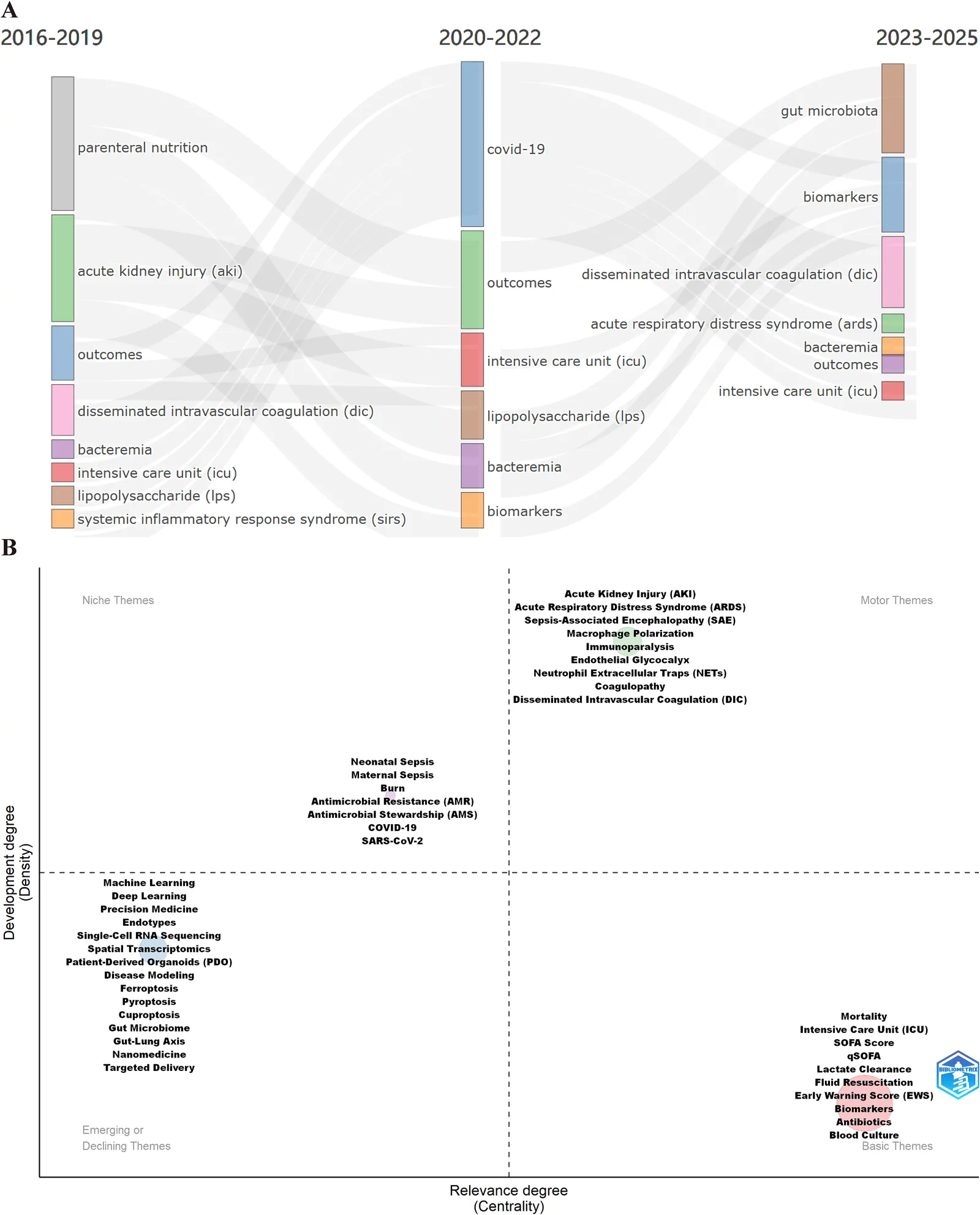
Keywords analysis. **(A)** Thematic evolution in the field of sepsis. **(B)** Thematic map: The horizontal axis represents mediating centrality, indicating the theme’s relevance to the field, while the vertical axis represents density, showing how well the theme has developed. Bubble size reflects the frequency of each theme, and different colors distinguish research categories. Quadrant 1 (top right) represents MOTOR themes, which are both important and well-developed. Quadrant 2 (top left) represents HIGHLY DEVELOPED and INDIVIDUAL themes—those that are well-developed but less relevant to the current domain. Quadrant 3 (bottom left) includes EMERGING OR DECLINING themes, which are underdeveloped and may be either gaining or losing relevance. Quadrant 4 (bottom right) represents BASIC and TRANSVERSAL themes, which are important to the field but not yet well-developed.

*Phase I (2016–2019): The Post-Sepsis-3 Transition*. Early research predominantly focused on traditional clinical interventions and acute organ dysfunction, reflected in large nodes such as “parenteral nutrition”, “AKI”, and “SIRS”. Notably, the “SIRS” node disappears after this phase (52).
*Phase II (2020–2022): The Pandemic Disruption*. The intermediate period was dominated by the emergence of “COVID-19”. This node acted as a central evolutionary hub, drawing research streams from earlier critical care topics. Concurrently, research interest consolidated around prognostic evaluation, reflected in the emergence of the “biomarkers” and “outcomes” clusters (53, 54).
*Phase III (2023–2025): Precision and the Microbiome*. In the current phase, research themes show increasing mechanistic specificity. The emergence of “gut microbiota” as a dominant terminal node—evolving directly from earlier “parenteral nutrition” and “lipopolysaccharide (LPS)” streams—reflects a shift toward gut-axis regulation and microbial homeostasis as inflammatory mediators of systemic sepsis (55–57). Meanwhile, “biomarkers” continues to expand, and complications such as “disseminated intravascular coagulation (DIC)” and “ARDS” have consolidated as core mechanistic research areas (58, 59).

The thematic map categorizes contemporary topics into four quadrants based on network centrality and density parameters (**Figure 7B**). Foundational clinical practices, such as the SOFA score and fluid resuscitation strategies, serve as basic and transversal themes. Organ-specific complications, including ARDS, AKI, and neutrophil extracellular traps (NETs), function as highly developed motor themes. Innovative technical frontiers, including ML, single-cell RNA sequencing (scRNA-seq), and ferroptosis pathways, cluster distinctly as emerging themes, whereas specialized domains, including neonatal sepsis and COVID-19, remain as localized niche themes.

## 4 Discussion

### 4.1 Global trajectory and paradigm shifts

This study characterizes the quantitative progression of the global sepsis research landscape over a 35-year period from 1991 to 2025. The documented publication volume conforms to a cubic polynomial growth model with an *R*^2^ = 0.9841, reflecting a structural transition from descriptive epidemiology toward mechanism-directed research. These patterns show that while emerging economies have expanded their absolute publication output, institutions in North America and Europe retain the highest citation impact.

The co-cited reference networks reveals a clear transition in the underlying empirical evidence base. Early critical care frameworks relied on expert consensus and retrospective evaluations (Level 5 and Level 3), exemplified by the initial Sepsis-3 definitions and early SSC guidelines. These have been progressively supplemented by high-level RCTs and prospective cohort studies (Level 1 and Level 2) targeting specific therapeutic interventions. This shift reflects empirical maturation, from physiological consensus toward prospective validation of standardized care bundles.

Critically, these bibliometric transitions mirror a parallel evolution in inflammatory conceptualization. The initial dominance of Level 5 expert consensus—exemplified by Bone et al. (1992) and the SIRS criteria—reflected an era in which sepsis was primarily understood as a generalized pro-inflammatory state amenable to broad cytokine blockade. The progressive integration of Level 1 and Level 2 clinical evidence represents not merely methodological advancement, but a paradigm shift: the repeated failure of non-selective anti-cytokine strategies demonstrated that sepsis-induced immune dysregulation is a biphasic process in which hyperinflammation and immunosuppression co-exist and sequentially dominate (12, 16). This repositioning has shifted the therapeutic focus from broad inflammatory suppression toward precision immunomodulation targeting specific molecular checkpoints.

Author and journal co-citation patterns show significant thematic continuity. A stable core of investigators integrate classic pathophysiological principles with modern clinical trial methodologies. Beyond the geographical concentration of output in North American and European centers, longitudinal thematic mapping identifies a fundamental shift in how sepsis is understood clinically (**Figure 7A**). Sepsis is no longer managed as a generalized SIRS requiring broad macroscopic intervention. Contemporary approaches instead address it as a heterogeneous, organ-specific condition requiring precision immunomodulation, microenvironmental profiling, and targeted micro-ecological regulation.

### 4.2 Mapping knowledge domains and disciplinary cross-talk

Keyword co-occurrence networks and dual-map overlay analysis show that sepsis research operates as an integrated translational framework (**Figures 4**, **6A**). Bidirectional citation flows connect basic molecular immunology with multi-disciplinary clinical and intensive care management. Specialized intensive care journals disseminate granular clinical parameters, while high-impact general medical journals establish foundational diagnostic frameworks and multicenter RCT data. Citation bursts from 2021 to 2025 reflect a shift from macroscopic clinical descriptions toward organ-specific pathophysiology and causal inference methodologies (**Figure 6B**).

#### 4.2.1 Pathophysiological mechanisms and systemic cascades to organ dysfunction

Clusters 1 and 5 span the continuum from subcellular pathogenesis to systemic organ failure. LPS-mediated innate immune activation recruits macrophages and neutrophils, triggering secretion of pro-inflammatory cytokines and nitric oxide that drive oxidative injury and subsequent functional immunosuppression (17, 60–62). Current research has focused on the NLRP3 inflammasome as a central node in this inflammatory cascade (**Figure 6B**). NLRP3 activation by PAMPs and DAMPs drives caspase-1-mediated maturation of IL-1β and IL-18, while gasdermin D-mediated pyroptosis amplifies the systemic inflammatory response through lytic cell death and extracellular DAMP release (13, 14). This mechanism directly links innate immune pattern recognition to the cytokine-driven endothelial injury and immunosuppressive phases that characterize sepsis progression. Concurrent research targets gut microbiota dysbiosis and cell-specific immune infiltration as additional regulatory mechanisms (13, 51).

These aberrant responses drive microvascular thrombosis and endothelial injury, contributing to DIC (63–65) and MODS (66). Thermal injury and major trauma share overlapping inflammatory cascades with pathogen-induced sepsis, serving as useful comparative models for understanding sterile systemic inflammatory responses (16, 67, 68).

#### 4.2.2 Etiological landscapes and challenges in vulnerable cohorts

Cluster 3 maps the microbiological profile of sepsis alongside specific demographic vulnerabilities. Core nodes representing bacteremia, pneumonia, and meningitis define primary clinical targets validated through blood cultures and specific pathogens such as *Staphylococcus aureus* (69, 70). This cluster includes neonatal sepsis and pregnancy-associated infections, where distinct microbiological profiles, altered pharmacokinetics, and unique physiological characteristics require specialized diagnostic and therapeutic approaches (71–73). Antimicrobial resistance intersects directly with this domain; optimizing empirical regimens against multi-drug resistant strains remains a critical determinant of survival in these vulnerable populations (74, 75).

#### 4.2.3 Clinical management and perioperative risk transitions

Clusters 4 and 2 together represent the primary clinical translational pathway. Initial evaluation involves rapid triage and shock differentiation using biochemical markers including C-reactive protein, procalcitonin, lactate, and IL-6 (76–79).

Following ICU admission (Cluster 2), clinical priorities shift toward mechanical ventilation and reducing 28-day mortality in patients with severe organ-specific complications, including septic cardiomyopathy and sepsis-associated encephalopathy (**Figure 6B**). Within perioperative medicine, surgical patients undergoing high-risk procedures such as major thoracic or oncological resections represent a distinct subpopulation. Post-operative infections can progress rapidly to systemic sepsis and secondary ARDS; integrating predictive strategies into surgical ICU (SICU) practice is therefore important for early intervention before irreversible multi-organ injury develops (80, 81).

#### 4.2.4 The digital frontier: algorithmic prediction and precision analytics

The co-clustering of AKI and ML in Cluster 6 reflects a shift from descriptive epidemiology toward predictive analytics. Standard biochemical markers such as serum creatinine show delayed kinetics relative to functional nephron injury, making SA-AKI a primary target for AI-driven predictive modeling in the ICU (82–84). ML frameworks trained on ICU time-series data and early biomarker signals can identify patients at risk of SA-AKI hours before overt clinical presentation (85), supported by recent citation bursts in Mendelian randomization and network pharmacology (**Figure 6B**). This approach supports a transition toward individualized critical care.

### 4.3 Emerging trends and future horizons

Longitudinal thematic evolution and quadrant mapping identify several emerging research priorities (**Figures 7A, B**). While foundational clinical parameters, including the SOFA score, fluid resuscitation protocols, and classical biochemical markers, remain the bedrock of acute clinical management (86–88), research focus has shifted toward mechanism-driven precision approaches. These computational findings highlight priorities at the intersection of data science, molecular immunology, and targeted pharmacology. Although bibliometric analyses identify rapidly expanding research domains, emerging trends should not be interpreted as restricting future investigations exclusively to these areas, as breakthrough discoveries may arise from currently underexplored mechanisms or pathways.

#### 4.3.1 AI-driven precision medicine and sepsis endotyping

The disappearance of the SIRS cluster and consolidation of biomarkers and outcomes nodes reflect the field’s definitive alignment with Sepsis-3 criteria, which prioritize infection-induced organ dysfunction over non-specific systemic inflammation (89). Traditional clinical scoring systems have limited predictive capacity for the heterogeneous sepsis population, and managing this heterogeneity increasingly requires computational approaches. Strategic mapping identifies a trend toward precision medicine using ML and deep learning (**Figure 7B**). Future studies should apply these frameworks to derive reproducible sepsis endotypes from multi-modal clinical data (90–92). Identifying distinct molecular or physiological subphenotypes can guide individualized fluid resuscitation and targeted therapy, directly linking clinical metrics with predictive models.

#### 4.3.2 Unraveling microenvironmental heterogeneity via multi-omics

Chronological mapping shows that the COVID-19 pandemic redirected research toward severe organ-specific pathology, including ARDS and DIC (**Figure 7A**). Understanding these injuries requires single-cell RNA sequencing and spatial transcriptomics (93, 94), which enable characterization of cell-to-cell communication networks—including macrophage polarization axes and endothelial glycocalyx degradation pathways—within the septic microenvironment (**Figure 7B**). Established animal models are increasingly supplemented by patient-derived organoids and bioengineered platforms (95, 96) that replicate human immunopathological phenotypes to support translationally relevant biomarker discovery.

#### 4.3.3 Novel regulated cell death pathways

Strategic mapping identifies a shift toward highly inflammatory RCD pathways—specifically ferroptosis, pyroptosis, and cuproptosis—as key mechanistic drivers (**Figure 7B**). These pathways contribute to the persistent DIC and ARDS networks identified in recent evolutionary phases (**Figure 7A**). Pyroptosis—driven by gasdermin D downstream of NLRP3 and caspase-4/5/11 activation—is particularly relevant to sepsis inflammatory biology: it amplifies the systemic inflammatory response through extracellular release of IL-1β and DAMPs, creating a feed-forward inflammatory loop that antimicrobial therapy alone cannot interrupt. Targeting this pathway at the inflammasome level therefore represents a mechanistically grounded anti-inflammatory strategy. Understanding the crosstalk between RCD pathways, immunoparalysis, and NET formation is essential for targeted therapeutic design. Molecular checkpoints such as gasdermin D in pyroptosis and GPX4 in ferroptosis represent actionable anti-inflammatory targets when antimicrobial stewardship fails to halt progressive organ injury (83, 97–99). Bibliometric momentum in this domain is high, but clinical translation requires prospective validation in registered trials.

#### 4.3.4 The gut-organ axis and targeted nanotherapeutics

A key structural change in the 2023–2025 phase was the shift from broad nutritional interventions toward targeted gut microbiota regulation (**Figure 7A**). This reflects a focus on gut dysbiosis and the gut-lung inflammatory axis as key mechanisms in sepsis-induced systemic failure (100, 101). Nanomedicine and targeted drug delivery systems aim to translate these findings into clinical practice (**Figure 7B**). Biocompatible nanoparticles designed to deliver anti-inflammatory agents or antimicrobials to inflamed endothelium or dysbiotic intestinal segments may overcome the limitations of systemic toxicity and restricted tissue penetration in severe sepsis management.

### 4.4 Implications for clinical practice, resource allocation, and policy

Translating these bibliometric findings into clinical policy requires balancing technological innovation with empirical validation, economic viability, and global equity. As structured within the clinical translational matrix (**Table 2**), emerging anti-inflammatory research domains are evaluated across scientific momentum, mechanistic validation status, and translational barriers—reflecting the field’s shift from broad inflammatory suppression toward targeted immunomodulatory interventions addressing specific nodes, including NLRP3 inflammasome activation, RCD cascades, and gut-axis immune dysregulation.

**Table 2:** Translational status, academic momentum, and bedside barriers in sepsis research.

Research domain & focus	Current evidence status & academic momentum	Clinical translation barriers & implications	Strategic future recommendations (policy & research)
Translational Impact & Evidence Evolution	• Robust paradigm shift from Level 5 expert consensus (e.g., SIRS/Sepsis-1/2) toward Level 1/2 clinical validation (Sepsis-3 criteria and protocolized care bundles).	• Operationalization Heterogeneity: Sepsis-3 standardized early diagnosis, but real-time bedside implementation of the SOFA score relies heavily on institutional resource availability and rapid clinical tracking.	• Policy: Dynamically update (SSC guidelines to incorporate organ-specific clinical endotypes. • Research: Prioritize Phase III clinical trials shifting focus from broad immunomodulation to precision targeted interventions.
Digital Intensive Care & Predictive Analytics	• Intense algorithmic momentum driven by active citation bursts: machine learning (strength: 149.72), artificial intelligence (strength: 50.77), and prediction models (strength: 49.49).	• Infrastructural Barriers & Opacity: Deploying AI models for real-time risk stratification (e.g., SA-AKI or 28-day mortality) demands massive electronic medical record infrastructure and risks clinical distrust due to “black-box” algorithms.	• Policy: Mandate rigorous safety, equity, and cost-effectiveness audits before hospital-wide AI deployment. • Research: Fund multi-institutional external validation of predictive algorithms to ensure cross-population reliability.
Global Health Equity & Geographic Disparities	• Pronounced publication asymmetry: United States dominance (30.32% output) contrast with a severe citation impact gap (China: 21.86 vs. Netherlands: 77.58 citations/publication); extreme scarcity of structured data from LMICs.	• Epidemiological Mismatch: The global sepsis mortality burden resides predominantly in resource-limited settings, yet available literature and international guidelines remain heavily biased toward high-income Western healthcare paradigms.	• Policy: Prioritize international funding for affordable point-of-care testing (POCT) and secure antibiotic supply chains in LMICs. • Training: Subsidize open-access publishing models for LMIC researchers and launch bidirectional global health fellowships.
Microenvironmental Heterogeneity & Pathophysiological Drivers	• Rapidly accelerating citation bursts highlighting mechanistic frontiers: Mendelian randomization (strength: 52.96), gut microbiota (strength: 48.13), and NLRP3 inflammasome (strength: 24.72).	• Mechanistic Translational Gap: The clinical focus is evolving from macroscopic fluid/vasopressor titration to micro-ecological and cellular regulation (e.g., the gut-lung axis), but high-fidelity human translational evidence remains limited.	• Research: Prioritize translational clinical trials targeting novel non-apoptotic RCD pathways (e.g., pyroptosis, ferroptosis) and expand causal inference via Mendelian randomization for robust drug repurposing.
Subpopulation Divergence & Antimicrobial Stewardship	• High-density thematic clustering and emergence of neonatal sepsis, maternal sepsis, and antimicrobial resistance (strength: 33.14) as highly relevant independent nodes.	• Guideline Extrapolation Risks: Standardized adult sepsis protocols fail to account for the unique microbiological spectrum, altered pharmacokinetics, and distinct physiological profiles of maternal and pediatric cohorts.	• Policy: Strictly enforce global, multi-tier antimicrobial stewardship policies to combat escalating resistance. • Research: Accelerate the development and validation of subpopulation-specific therapeutic bundles tailored exclusively to neonatal and pregnancy-associated sepsis.

AI, artificial intelligence; LMICs, low- and middle-income countries; NLRP3, NLR family pyrin domain containing 3; POCT, point-of-care testing; RCD, regulated cell death; SA-AKI, sepsis-associated acute kidney injury; SOFA, sequential organ failure assessment; SSC, surviving sepsis campaign.

#### 4.4.1 Clinical practice and strategic resource allocation

While rapid triage and protocolized resuscitation remain foundational, the rapid adoption of ML and AI creates resource-allocation challenges. Deploying algorithmic monitoring for severe complications such as SA-AKI requires substantial investment in electronic health record infrastructure and data science personnel. Given limited multi-center prospective validation, lack of interpretability, and risks of alert fatigue, widespread implementation requires rigorous cost-effectiveness analysis. Consequently, resource-constrained facilities should prioritize standard biochemical markers—specifically procalcitonin kinetics and lactate clearance—and adherence to established guidelines, rather than adopting complex predictive frameworks that lack local clinical validation.

#### 4.4.2 Addressing disparities in global health equity

Geographical metadata reveal an important epidemiological paradox: the global sepsis research agenda is heavily concentrated in high-income settings, whereas the highest mortality burden remains in resource-limited regions. Although China ranks second globally in publication volume, its average citation impact (21.86) is substantially lower than that of established Western leaders such as the Netherlands (77.58), reflecting a divergence between publication scale and translational integration. Addressing this imbalance requires policy interventions that extend beyond publication metrics. Global health initiatives should balance funding between advanced omics research in high-income nations and foundational critical care infrastructure in low- and middle-income countries (LMICs), prioritizing low-cost point-of-care diagnostics, stable antimicrobial supply chains, and open-access publication support to improve the visibility of research from underserved settings.

#### 4.4.3 Directing future research investments

Future research investment should shift from descriptive analyses toward mechanistic and causal frameworks. Strategic priorities include targeting the NLRP3 inflammasome and gut microbiota dysbiosis, and applying causal inference methods such as Mendelian randomization to establish robust relationships needed for drug repurposing trials. The distinct epidemiological clustering of maternal and neonatal sepsis supports the need for dedicated prospective trials to develop subpopulation-specific diagnostic and management protocols, integrated with structured antimicrobial stewardship programs to address the growing threat of multi-drug resistance.

### 4.5 Limitations and future horizons

This study has several limitations. First, although a comprehensive multi-database search was conducted, studies indexed exclusively in regional or specialized repositories may have been omitted. Restriction to English-language publications introduces geographical bias, underrepresenting evidence from non-Anglophone settings and compounding data scarcity from LMICs. Second, the human-centered filtering protocol excluded preclinical and *in vitro* studies to preserve clinical relevance. This may underrepresent early-stage mechanistic findings in RCD pathways and multi-omics research that lack human clinical validation. To reconcile this filtering protocol with the high density of molecular targets discussed, it is critical to note that contemporary clinical sepsis studies increasingly integrate patient-derived translational omics. Consequently, deep mechanistic nodes—such as the NLRP3 inflammasome, gasdermin D-driven pyroptosis, and GPX4-mediated ferroptosis—frequently co-occur with high citation momentum directly within our clinically filtered dataset, representing the field’s active bedside-to-bench translation rather than an analytical discrepancy. Third, citation accumulation is inherently retrospective and may understate the immediate impact of work published in the 2023–2025 period. Algorithmic burst detection mitigates this lag but cannot fully compensate for it. Finally, bibliometric frameworks depend on metadata consistency; variations in author keyword selection can introduce minor inconsistencies in topological clustering despite rigorous data standardization.

Future studies should integrate advanced natural language processing (NLP) to move beyond metadata constraints. Incorporating altmetrics—including policy citations and guideline adoptions—would provide a broader view of how bibliometric trends influence clinical decision-making. Transitioning from abstract-based to full-text NLP analysis would yield more granular insights into clinical endpoints such as 28-day mortality and real-world ML model performance. Ultimately, correlating computational literature trends with real-world ICU databases would help validate whether scholarly momentum translates into measurable improvements in patient outcomes and global health equity.

## 5 Conclusion

This 35-year systematic mapping of 106,884 publications delineates the structural evolution of inflammatory research paradigms in sepsis, documenting a transition from SIRS-based pro-inflammatory consensus toward recognition of immune dysregulation—encompassing biphasic hyperinflammation and immunosuppression—as the central pathophysiological framework. This shift is reflected in the progressive integration of high-level clinical trial evidence into the co-citation network, and in the emergence of precision immunomodulatory strategies as the defining research frontier of the Sepsis-3 era.

Keywords with citation burst, thematic evolution and thematic mapping identify the NLRP3 inflammasome, regulated cell death pathways (pyroptosis, ferroptosis), and the gut-lung inflammatory axis as the most active emerging anti-inflammatory research targets. These domains represent mechanistically grounded opportunities for endotype-specific therapeutic interventions targeting dysregulated immune pathways, moving beyond the non-selective cytokine blockade strategies that characterized earlier sepsis research.

At the same time, the global research landscape remains structurally imbalanced: high-citation output is concentrated in North American and European institutions while the greatest mortality burden falls on resource-limited settings. The clinical translational matrix provides a framework for reconciling these priorities, guiding investment in both precision immunomodulatory research and equitable critical care capacity-building. Integrating AI-driven endotyping, multi-omics profiling, and personalized immunomodulatory strategies that account for both hyperinflammatory and immunosuppressive phenotypes—alongside reinforcement of foundational care and global health equity—represents a key direction for the next decade of sepsis research.

## Data Availability

The original contributions presented in the study are included in the article/**Supplementary Material**. Further inquiries can be directed to the corresponding author/s.
