# Supplementary material for: Thirty-five years of evolving inflammatory paradigms in sepsis (1991–2025): a comprehensive mapping of immune dysregulation research, anti-inflammatory targets, and translational barriers
Source: Front Immunol. 2026 Sep 21;17:1892665. doi: 10.3389/fimmu.2026.1892665 (PMC13635366; doi:10.3389/fimmu.2026.1892665)
Supplement: Supplementary file 1 [file DataSheet1.pdf]

Supplementary Material to:

Thirty-Five Years of Evolving Inflammatory Paradigms in Sepsis (1991–2025): A Comprehensive Mapping of Immune Dysregulation Research, Anti-Inflammatory Targets, and Translational Barriers

Hao-Han Rao[1], Feng Guo[1], Jie Tian[1], Chun-Jian Zuo[1], Huan-Wen Chen[1*]

[1]Department of Thoracic Surgery, The First Affiliated Hospital of Chongqing Medical University, Chongqing 400016, China.

*Corresponding author: Dr. Huan-Wen Chen, Department of Thoracic Surgery, The First Affiliated Hospital of Chongqing Medical University, No.1, Youyi Road, Yuzhong District, Chongqing 400016, China. Email: coolstarchw9527@163.com. Fax: +86-23-88955807. Phone: +86-23-88955807

Contents

- **Supplementary Figure 1: Literature identification, screening, and dataset construction workflow for bibliometric analysis.**
- **Supplementary Table 1: The top 10 countries and organizations related to the research of sepsis.**
- **Supplementary Table 2: The top 10 journals and co-cited journals related to the research of sepsis.**
- **Supplementary Table 3: The top 10 authors and co-cited authors related to the research of sepsis.**
- **Supplementary Table 4: The top 50 keywords related to the research of sepsis.**

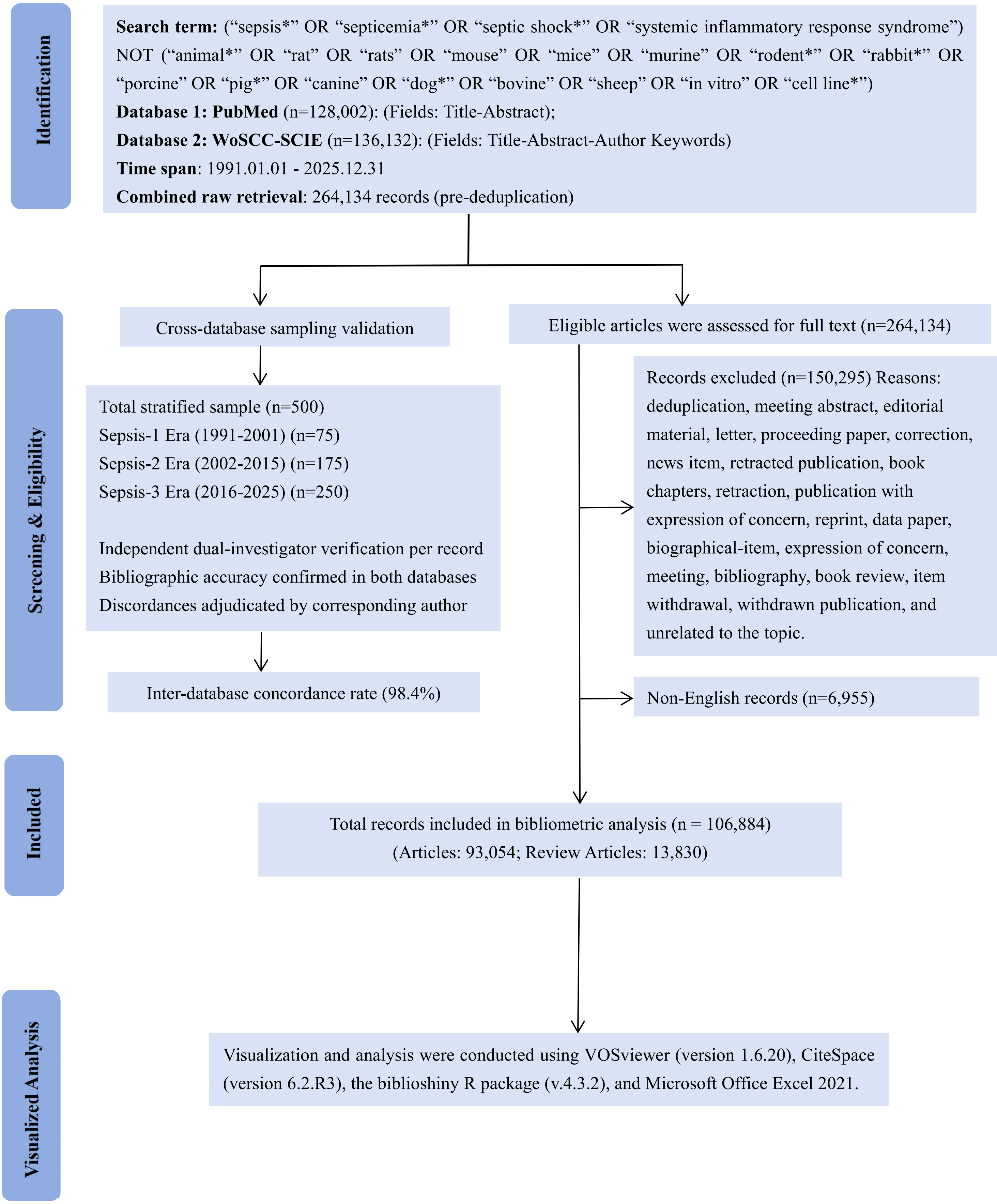

Supplementary Figure 1: Literature identification, screening, and dataset construction workflow for bibliometric analysis.

Supplementary Table 1: The top 10 countries and organizations related to the research of sepsis.

Rank	Country	Counts	Citations	Average Citation/ Publications	Organization	Counts	Citations
1	United States	32406	1921734	59.30	University of Pittsburgh (United States)	1356	167521
2	China	18679	408408	21.86	University of Toronto (Canada)	1094	102866
3	United Kingdom	7770	515644	66.36	University of Pennsylvania (United States)	1005	63588
4	Germany	7363	481762	65.43	University of Michigan (United States)	984	83358
5	Italy	5196	271051	52.17	Harvard University (United States)	959	94821
6	France	5076	390811	76.99	University of Amsterdam (Netherlands)	880	69745
7	Canada	4493	357624	79.60	University of Washington (United States)	858	65694
8	Japan	4323	167930	38.85	Harvard Medical School (United States)	813	37023
9	Australia	3857	257977	66.89	Zhejiang University (China)	796	19216
10	Netherlands	3486	270449	77.58	Emory University (United States)	757	82084

Supplementary Table 2: The top 10 journals and co-cited journals related to the research of sepsis.

Rank	Journal	Counts	Citations	IF[a]	Q[b]	Co-cited journal	Co-citation	IF[a]	Q[b]
1	Critical Care Medicine	2391	247955	6	1	Critical Care Medicine	154264	6	1
2	PLOS ONE	1670	51139	2.6	2	New England Journal of Medicine	87080	78.5	1
3	Shock	1604	59135	2.9	1	Intensive Care Medicine	68341	22.1	1
4	Critical Care	1591	113285	9.3	1	JAMA: The Journal of the American Medical Association	67118	55	1
5	Scientific Reports	1091	19320	3.9	1	Critical Care	66625	9.3	1
6	Intensive Care Medicine	1078	103212	22.1	1	Journal of Immunology	53860	3.4	2
7	Frontiers in Immunology	1004	33583	5.9	1	Lancet	51684	88.5	1
8	Journal of Critical Care	827	23229	2.9	2	PLOS ONE	44049	2.6	2
9	BMC Infectious Diseases	762	13778	3	2	Journal of Biological Chemistry	43843	3.9	2
10	Medicine	717	10605	1.4	2	Clinical Infectious Diseases	41192	7.3	1

[a]The impact factor of the journal are obtained from Journal Citation Reports 2024.

[b]The quartile of the journal are obtained from Journal Citation Reports 2024.

Supplementary Table 3: The top 10 authors and co-cited authors related to the research of sepsis.

Rank	Author	Counts	Citations	H-index	Co-cited Authors	Citations
1	Jean-Louis Vincent	425	82872	123	M. Singer	13020
2	Rinaldo Bellomo	353	51963	87	J. L. Vincent	12643
3	Tom Van Der Poll	241	40766	71	R. C. Bone	11635
4	Claudio Ronco	189	18560	55	R. S. Hotchkiss	8460
5	Ping Wang	168	7375	55	R. P. Dellinger	7701
6	John A. Kellum	157	21480	76	M. M. Levy	5799
7	Peter Pickkers	140	10457	58	D. C. Angus	5264
8	Wei Zhang	140	2850	48	D. Annane	4883
9	Djillali Annane	138	39925	65	W. A. Knaus	4416
10	Evangelos J. Giamarellos-Bourboulis	134	5407	42	A. Kumar	3941

Supplementary Table 4: The top 50 keywords related to the research of sepsis.

Rank	Keywords	Counts	Rank	Keywords	Counts
**1**	Sepsis	27063	**26**	Multiple Organ Dysfunction Syndrome (MODS)	863
**2**	Septic Shock	6371	**27**	Diagnosis	812
**3**	Intensive Care Unit (ICU)	5075	**28**	Macrophage	797
**4**	Mortality	4618	**29**	C-Reactive Protein	772
**5**	Outcomes	4269	**30**	Trauma	757
**6**	Inflammation	3964	**31**	Oxidative Stress	748
**7**	Newborn	2850	**32**	IL-6	724
**8**	Infections	2838	**33**	Machine Learning	687
**9**	Lipopolysaccharide (LPS)	2454	**34**	Meta-Analysis	676
**10**	Acute Kidney Injury (AKI)	2241	**35**	Disseminated Intravascular Coagulation (DIC)	671
**11**	Acute Respiratory Distress Syndrome (ARDS)	2072	**36**	Nitric Oxide	635
**12**	Pediatrics	1972	**37**	Surgery	622
**13**	Cytokines	1937	**38**	Immunosuppression	576
**14**	Bacteremia	1904	**39**	Cancer	548
**15**	Biomarkers	1777	**40**	Staphylococcus Aureus	528
**16**	Systemic Inflammatory Response Syndrome (SIRS)	1768	**41**	Emergency Department	525
**17**	Neonatal Sepsis	1351	**42**	Innate Immunity	509
**18**	Risk Factors	1316	**43**	Blood Culture	501
**19**	Covid-19	1269	**44**	Neutrophils	491
**20**	Procalcitonin	1141	**45**	Pregnancy	488
**21**	Complications	1110	**46**	Lactate	466
**22**	Antibiotics	1048	**47**	Meningitis	452
**23**	Apoptosis	935	**48**	Antimicrobial Resistance	449
**24**	Pneumonia	923	**49**	Burn	447
**25**	Epidemiology	902	**50**	Mechanical Ventilation	444
